# A Rare Nephrotic Syndrome Related to Chronic Lymphocytic Leukemia: Focal Segmental Glomerulosclerosis

**DOI:** 10.7759/cureus.31545

**Published:** 2022-11-15

**Authors:** Volkan Karakus, Unal Atas, Sahnura Uzuntas, Yelda Dere, Ibrahim Meteoglu

**Affiliations:** 1 Department of Hematology, Antalya Training and Research Hospital, Antalya, TUR; 2 Department of İnternal Medicine, Alanya Alaaddin Keykubat University, Antalya, TUR; 3 Department of Pathology and Laboratory Medicine, Muğla Sıtkı Koçam University, Muğla, TUR; 4 Department of Pathology, Adnan Menderes University, Aydın, TUR

**Keywords:** autoimmune disease and cancer, immunosuppression, glomerulonephritis (gn), focal segmental glomerulosclerosis (fsgs), chronic lymphocytic leukemia (cll)

## Abstract

Chronic lymphocytic leukemia (CLL) is a hematological disease characterized by the proliferation of monoclonal B-lymphocytes. Although autoimmune complications such as autoimmune hemolytic anemia and immune thrombocytopenia are common in CLL patients, nonhematological autoimmune complications are rather rare. The most common renal involvements are membranoproliferative glomerulonephritis and minimal change disease. Focal segmental glomerulosclerosis (FSGS) is predominantly associated with Hodgkin's lymphoma among hematological malignancies. FSGS associated with CLL is rarely reported in the literature, with a poor understanding of the common pathophysiology and a very limited experience with this co-occurrence. Although Rai Stage 1/Binet Stage B CLL, our 61-year-old case, who was diagnosed with secondary FSGS, which is a very rare complication, was treated with fludarabine, cyclophosphamide, and rituximab (FCR) combination. Following the treatment, a complete response was achieved about CLL, and the patient, whose renal findings recovered, is in remission and under follow-up for six years. Although the mechanisms between CLL and autoimmune complications are not fully elucidated, it is usually related to immune disorders like an abnormal T-cell response and polyclonal antibody production. While FSGS is very rare in lymphoma, its co-existence with CLL is reported only in a limited number of case reports. Steroids may be used in these patients; however, in cases not responding to steroids, treatment of the underlying CLL is required.

## Introduction

Chronic lymphocytic leukemia (CLL) is characterized by the proliferation and infiltration of the monoclonal B-lymphocytes. Although autoimmune complications are encountered approximately in one-fourth of all CLL cases, nonhematological autoimmunity disorders are very rare [1]. Glomerulonephritis, angioedema, and paraneoplastic pemphigus are nonhematological, CLL-associated autoimmune disorders [2]. Glomerulonephritis is seen rarely in the course of hematological malignancies. Although mostly the minimal change disease is seen, it was reported that it usually accompanies Hodgkin's disease [3]. Focal segmental glomerulosclerosis (FSGS) is a kidney disease that is accompanied by hypertension, progressive renal failure, and proteinuria. Although certain important hypotheses were introduced in primary ones, their etiologies are not elucidated yet [4]. FSGS is the most rarely reported glomerulonephritis among the renal lesions related to hematological malignancies, and, even though in a few cases, it has been mostly reported in patients with Hodgkin's lymphoma. Membranoproliferative glomerulonephritis and membranous glomerulonephritis are frequently reported during CLL [3]. However, considering the literature, CLL-associated FSGS is reported in a limited number of case reports, and there is a little experience [3,5]. In this paper, we presented a case treated due to secondary FSGS, which emerged in the early stage of the CLL course as a very rare condition.

## Case presentation

An asymptomatic 61-year-old female patient is referred with the findings of lymphocytosis and lymphadenopathy, which were detected during a routine medical examination. During the physical examination, no additional findings were determined except for the about 2 cm × 2 cm pathological lymphadenopathies in the neck and axillary region. CBC revealed leukocytes 18,200 mm^-3^; lymphocytes 12,700 mm^-3^, platelets 191,000 mm^-3^, and hemoglobin 12.6 mg/dL. Serum levels of immunoglobulins and autoimmune markers were within normal range, and viral markers and the Coombs test were negative. The peripheral smear examination revealed increased small, similar-type mature lymphoid cells and basket cells. The flowcytometric examination of peripheral blood showed that CD5+, CD19+, and CD23+ co-occurrence rate was 79% in the 82% lymphoid population, and the patient was diagnosed with Rai Stage 1/Binet Stage B CLL, depending on the present findings. We decided on a follow-up without pharmacotherapy and no additional laboratory change except for the increase in the lymphocyte count (18,000 mm^-3^) was observed during the 20-month follow-up. However, at the 20th month of follow-up, the patient developed pretibial edema. As the examinations displayed albumin-predominant proteinuria (1.2 g/24 hours) and a serum albumin level of 3.6 g/dL, a renal biopsy was carried out. Global sclerosis was determined in the 6 of 11 glomeruli examined in the biopsy material, and segmental sclerosis was noticed in other glomeruli. Furthermore, mild chronic inflammation, moderate fibrosis in the interstitial space, prominent medial hyalinization in vessels, and atrophy in the tubules were noticed. Direct immunofluorescent examination showed that there was no accumulation of IgA, IgG, IgM, C3, C1q, C4, fibrinogen, and kappa and lambda chains and that amyloid was negative. During two months, which passed with examination and tests, the creatinine level of the patient was within normal limits while proteinuria increased to 3.4 g/24 hours. Although the patient was known to have Rai Stage 1/Binet Stage B, as the patient was diagnosed with secondary FSGS, she was treated with a six-cycle treatment of fludarabine, cyclophosphamide, and rituximab (FCR) combination. Proteinuria dropped to 1.1 g/24 hours after the first cycle and disappeared after the third cycle. In the sixth year of the treatment, the patient continues to be followed up with a full response in respect of CLL and without any renal problems, including proteinuria.

## Discussion

In this paper, we presented an early stage CLL patient who was treated due to the development of FSGS and had a complete response to CLL treatment and recovery in renal functions after the treatment. Although membranoproliferative glomerulonephritis and membranous glomerulonephritis are more common in CLL patients, FSGS concomitant to CLL was reported only in five case reports [3,5].

The mechanisms between CLL and autoimmune complications are not fully elucidated, but it is usually associated with immune system disorders such as abnormal T-cell response and polyclonal antibody production [6]. A hypothesis about the relationship between glomerular disease and the accumulation of the circulating monoclonal immunoglobulins or light chains is proposed. As it seems, there is no established correlation between the CLL disease stage and glomerulonephritis [3].

Steroids are an outstanding treatment choice for FSGS, but specific immunosuppressive agents like cyclosporine may be used in some resistant cases. Alkylating agents are also preferred in steroid-resistant cases [7]. Regarding the CLL-related FSGS cases in the literature, as far as it is determined, one case was treated with chlorambucil + prednisolone, two cases with cyclophosphamide + prednisolone, and one case with FCR, and the treatments were reported to be effective [5,8]. Currently, FCR combination therapy is still an acceptable first-line treatment for young CLL patients with immunoglobulin heavy-chain variable region gene (IGHV) mutation, without 17p deletion or TP53 mutation [9,10]. The effects of fludarabine on lymphocytes are well known, and they actively control the disease [11]. However, the potential for the development of fludarabine-mediated autoimmune cytopenia may limit its use in the treatment of autoimmune complications. Available evidence indicates that the risk of developing autoimmune cytopenia after purine analog exposure is not higher than in other agents. Furthermore, the evaluation of the relevant clinical trials showed that the addition of fludarabine to other agents such as cyclophosphamide and rituximab might decrease the risk of autoimmune complications [9-11]. Besides, although there is a risk of FSGS related to fludarabine, no additional glomerulopathy problem was encountered in cases treated with fludarabine due to FSGS. Anti-CD20 molecules act by selectively lysing lymphocytes during the treatment of CLL, and it has been shown that they increase the effectiveness of the treatment [12]. Besides, it was shown that rituximab treatment was effective in some autoimmune complications of CLL [6]. Some FSGS cases treated with regimes not containing rituximab did not respond to the treatment [1,4]. In line with all these findings, after the assessment of the limited number of FSGS-related CLL cases, FCR treatment was preferred as the patient was younger than 65 years and had not undergone any treatment before.

## Conclusions

B-cells play an important role in immune cells via antigen processing and presentation, interaction with autoreactive T-cells and antigen-presenting cells, and cytokine secretion. It is believed that FCR treatment leads to B-cell destruction and plays an important role in the decrease in antibody levels, which are linked to the pathophysiology, by decreasing T-cell activation and T-cell induction. It is suggested that the FCR regime, which is an effective treatment in CLL, may be also effective in both FSGS and CLL-related FSGS.

## References

[1] Hamblin TJ (2006). Autoimmune complications of chronic lymphocytic leukemia. Semin Oncol.

[2] Hamblin TJ (2009). Non-hemic autoimmunity in CLL. Leuk Res.

[3] Mallouk A, Pham PT, Pham PC (2006). Concurrent FSGS and Hodgkin's lymphoma: case report and literature review on the link between nephrotic glomerulopathies and hematological malignancies. Clin Exp Nephrol.

[4] Bose B, Cattran D (2014). Glomerular diseases: FSGS. Clin J Am Soc Nephrol.

[5] Arampatzis S, Giannakoulas N, Liakopoulos V (2011). Simultaneous clinical resolution of focal segmental glomerulosclerosis associated with chronic lymphocytic leukaemia treated with fludarabine, cyclophosphamide and rituximab. BMC Nephrol.

[6] Hodgson K, Ferrer G, Montserrat E, Moreno C (2011). Chronic lymphocytic leukemia and autoimmunity: a systematic review. Haematologica.

[7] Korbet SM, Schwartz MM, Lewis EJ (1994). Primary focal segmental glomerulosclerosis: clinical course and response to therapy. Am J Kidney Dis.

[8] Rosado MF, Morgensztern D, Abdullah S, Ruiz P, Lossos IS (2004). Chronic lymphocytic leukemia-associated nephrotic syndrome caused by focal segmental glomerulosclerosis. Am J Hematol.

[9] Rai KR, Stilgenbauer S (2022). Selection of Initial Therapy for Symptomatic or Advanced Chronic Lymphocytic Leukemia. In: Up To Date Hematology.

[10] Kipps TJ, Stevenson FK, Wu CJ (2017). Chronic lymphocytic leukaemia. Nat Rev Dis Primers.

[11] Gassner FJ, Weiss L, Geisberger R (2011). Fludarabine modulates composition and function of the T cell pool in patients with chronic lymphocytic leukaemia. Cancer Immunol Immunother.

[12] Strugov V, Stadnik E, Virts Y, Andreeva T, Zaritskey A (2018). Impact of age and comorbidities on the efficacy of FC and FCR regimens in chronic lymphocytic leukemia. Ann Hematol.

